# A Robot Hand Testbed Designed for Enhancing Embodiment and Functional Neurorehabilitation of Body Schema in Subjects with Upper Limb Impairment or Loss

**DOI:** 10.3389/fnhum.2015.00026

**Published:** 2015-02-19

**Authors:** Randall B. Hellman, Eric Chang, Justin Tanner, Stephen I. Helms Tillery, Veronica J. Santos

**Affiliations:** ^1^Biomechatronics Laboratory, Department of Mechanical and Aerospace Engineering, Arizona State University, Tempe, AZ, USA; ^2^Biomechatronics Laboratory, Department of Mechanical and Aerospace Engineering, University of California Los Angeles, Los Angeles, CA, USA; ^3^SensoriMotor Research Group, School of Biological and Health Systems Engineering, Arizona State University, Tempe, AZ, USA

**Keywords:** amputee, body schema, embodiment, hand, neurorehabilitation, phantom limb pain, robotic, upper limb

## Abstract

Many upper limb amputees experience an incessant, post-amputation “phantom limb pain” and report that their missing limbs feel paralyzed in an uncomfortable posture. One hypothesis is that efferent commands no longer generate expected afferent signals, such as proprioceptive feedback from changes in limb configuration, and that the mismatch of motor commands and visual feedback is interpreted as pain. Non-invasive therapeutic techniques for treating phantom limb pain, such as mirror visual feedback (MVF), rely on visualizations of postural changes. Advances in neural interfaces for artificial sensory feedback now make it possible to combine MVF with a high-tech “rubber hand” illusion, in which subjects develop a sense of embodiment with a fake hand when subjected to congruent visual and somatosensory feedback. We discuss clinical benefits that could arise from the confluence of known concepts such as MVF and the rubber hand illusion, and new technologies such as neural interfaces for sensory feedback and highly sensorized robot hand testbeds, such as the “BairClaw” presented here. Our multi-articulating, anthropomorphic robot testbed can be used to study proprioceptive and tactile sensory stimuli during physical finger–object interactions. Conceived for artificial grasp, manipulation, and haptic exploration, the BairClaw could also be used for future studies on the neurorehabilitation of somatosensory disorders due to upper limb impairment or loss. A remote actuation system enables the modular control of tendon-driven hands. The artificial proprioception system enables direct measurement of joint angles and tendon tensions while temperature, vibration, and skin deformation are provided by a multimodal tactile sensor. The provision of multimodal sensory feedback that is spatiotemporally consistent with commanded actions could lead to benefits such as reduced phantom limb pain, and increased prosthesis use due to improved functionality and reduced cognitive burden.

## Introduction

Upper limb impairment or loss can be caused by a multitude of factors including disease, trauma, surgery, and brain infarction (Harris, 1999; Dickstein and Deutsch, 2007). There is a 50–80% chance that when one loses a limb, an incessant pain called “phantom limb pain” will remain after amputation (Nikolajsen and Jensen, 2001). The pain can occur immediately after trauma or may take months to years to develop. The root cause of phantom limb pain is not well understood and may be due to an irritation of nerve endings, a central remapping of sensations that results in misinterpreted activations of pain neurons, or the mismatch of motor commands and visual feedback that are then interpreted as pain (Ramachandran and Hirstein, 1998). Even in the absence of severe pain, amputees often refer to their missing limbs as feeling paralyzed in an uncomfortable or cramped position. Patients often experience depression due to the pain and discomfort that is degrading their quality of life. In this work, we discuss potential clinical benefits to upper limb amputees that could arise from the confluence of known concepts such as mirror visual feedback (MVF) and the “rubber hand” illusion, and new technologies such as neural interfaces for artificial sensory feedback and highly sensorized robot hand testbeds, such as the “BairClaw” presented here.

### Non-invasive, vision-based therapies for pain disorders and paralysis

Mirror visual feedback was introduced in 1992 as a non-invasive technique to treat phantom limb pain due to amputation, and paralysis due to stroke (Ramachandran and Altschuler, 2009). A mirror or virtual environment is used to provide a visualization of one’s missing or hidden impaired limb by reflecting the movement of the contralateral unimpaired limb. Despite inaction by the impaired limb, this technique results in activation of regions of the brain corresponding to the lost or impaired limb. When MVF was first examined over 20 years ago, pain disorders and paralysis were believed to be untreatable. Since then, MVF has been used to treat complex regional pain syndrome and peripheral nerve damage. Even though MVF is not a panacea, it has been shown to be an effective form of therapy for phantom limb pain (Stevens and Stoykov, 2003; Darnall, 2009). Any positive treatment can have a large impact considering the high occurrence of phantom limb pain in amputees, and the fact that strokes are the leading cause of long-term disability (Go et al., 2014).

Graded motor imagery (GMI) is a variation of MVF that has had success in reducing pain and discomfort associated with pain and movement problems. GMI consists of three steps: left/right discrimination, motor imagery exercises, and mirror therapy (Moseley, 2006; Johnson et al., 2012). When first starting GMI treatment, left/right discrimination is the primary focus because it has been shown that individuals with chronic pain are less accurate and/or slower in determining whether an image is of a left or right limb compared to healthy individuals (Schwoebel et al., 2001). Difficulty in determining laterality reflects the lack of a strong body schema. Motor imagery exercises such as imagining hand movements aid in increasing activity of motor cortical neurons that is involved with observed, imagined, or executed movements (Rizzolatti and Craighero, 2004). Strengthening of the body schema through left/right discrimination and explicit motor imagery creates a foundation upon which subsequent mirror therapy can be most effective (di Pellegrino et al., 1992; Rizzolatti et al., 1996; Priganc and Stralka, 2011). Through the use of GMI and sensory feedback to the phantom limb, it should be possible for a neuroprosthetic or robotic system to be incorporated into one’s body schema, which could aid in the treatment of phantom limb pain and improve functional performance with prosthesis.

### The “rubber hand” illusion

Studied often, the rubber hand illusion phenomenon illustrates the interactions among vision, touch, and proprioception as they relate to the body’s self-identification (Botvinick and Cohen, 1998). The illusion is created by hiding the subject’s hand out of view and then placing a rubber hand in its place. Both the subject’s hand and the rubber hand are brushed simultaneously. After some time, the subject can develop a sense of ownership with the rubber hand and disassociate from his/her native hand, reporting the feeling of brush strokes when only the rubber hand is brushed (Botvinick and Cohen, 1998). Subjects also experience “proprioceptive drift,” which describes the phenomenon in which subjects report the location of his/her native hand as being closer to the rubber hand than the native hand’s actual location. That is, the proprioceptive percept of the subject’s native hand has “drifted” toward the rubber hand.

Neuroplasticity and the ability to incorporate an artificial limb into one’s body schema date back to studies from 1937. Early work on the Aristotle illusion examined localization errors in perceived tactile stimuli when an object was touched simultaneously by the outer regions of two crossed fingers (Tastevin, 1937). For example, simultaneous contact of one’s nose with the radial aspect of the index finger and ulnar aspect of the middle finger can result in the perception that one has two noses. Recent studies have further demonstrated a link between one’s body schema and the physiological self, and how expressions of this link manifest themselves in measurable physiological changes. It has been hypothesized that increased ownership of an artificial limb disrupts regulation of certain aspects of the native limb. Interestingly, as an artificial upper limb becomes accepted into one’s body schema, the temperature of the native limb decreases (Moseley et al., 2008). Other experiments have shown that, through the rubber hand illusion, subjects’ immunological responses can be altered. The immune system’s primary goal is to discriminate self from non-self in order to protect the body from foreign organisms. In one such experiment, the response to a topically applied histamine was altered during the rubber hand experiment; welt size was larger for the hidden native limb when the illusion was in effect (Barnsley et al., 2011). The ability to manipulate the physiological response of the body through a visual illusion leads one to believe that the addition of congruent proprioceptive and touch feedback could accelerate the incorporation of neuroprosthesis into one’s body schema.

In a 2012 rubber hand illusion experiment, biological fingerpads were subjected to vibrotactile stimulation while a rubber hand was stroked or tapped (D’Alonzo and Cipriani, 2012). When vibrotactile stimuli were synchronized with the visual feedback, subjects developed a sense of ownership of the fake hand, despite the sensory substitution and modality mismatched nature of the sensory feedback to the biological hand. Recently, a single-digit version of the rubber hand illusion was conducted with a tactile sensor (Hartmann et al., 2014). The fingertip sensor was stroked and pressed in different regions in each subject’s view while the subject’s native hidden forearm was electrocutaneously stimulated according to changes in the tactile sensor data. Preliminary findings showed that subjects’ skin temperature decreased slightly in the native limb and proprioceptive drift resulted, as would be expected when the illusion is successful. Interestingly, even though some subjects indicated a lack of embodiment of the green-colored fingertip sensor, and sensory substitution methods were employed, researchers still observed physiological signs of a subconscious incorporation of the artificial finger into the body schema.

### Proprioceptive and exteroceptive feedback for amputees

Prior work suggests that tactile and proprioceptive inputs are encoded simultaneously in unimpaired individuals (Rincon-Gonzalez et al., 2012). For instance, non-weight bearing contact of the fingertip against a surface can improve the accuracy of perceived posture. In turn, limb posture can significantly change the cortical response to identical tactile stimuli (Rincon-Gonzalez et al., 2011). Sensory feedback mappings are clearly a function of both proprioceptive and exteroceptive information. Ongoing efforts to artificially produce conscious perceptions of phantom limb posture, motion, and contact with objects could be accelerated if proprioceptive and exteroceptive information could be provided to an amputee simultaneously and in an intuitive manner.

#### Natural sensory feedback from the residual limb

The body-powered, cable-driven prosthesis is still a popular choice for many amputees. While rejection rates remain somewhat high for powered myoelectric prostheses (35 and 23% for children and adults, respectively) (Biddiss and Chau, 2007a), many amputees prefer the speed of control and immediate natural sensory feedback obtained via extended physiological proprioception. During operation, cable excursion and stiffness can be sensed through the prosthesis socket as well as through the body harness (e.g., standard figure eight harness or cutaneous anchor adhered directly to the skin) (Williams, 2011). Although direct joint movement information is not available, body-powered prosthesis users are able to learn how to use this extended form of proprioception for the grasp and manipulation of objects. It has been shown that functional performance with body-powered prostheses can be further improved when the prosthesis is designed with extended physiological proprioception in mind (Doubler and Childress, 1984).

#### Methods for the provision of artificial sensory feedback

Communicating proprioceptive and exteroceptive information to amputees remains a grand challenge. Non-invasive sensory substitution methods using vibrotactile or electrotactile stimuli can be used to provide feedback, but the feedback is typically non-intuitive or difficult to scale to a multitude of simultaneous signals (Kaczmarek et al., 1991). For example, vibrations can be applied to a residual limb in relation to prosthesis grip force, but the amputee must learn this non-intuitive mapping. This may suffice for a single channel of information, but additional non-intuitive vibratory feedback that is simultaneously applied to other regions of the body will likely increase the cognitive burden on the user. In different studies, subjects often reported that feedback provided via sensory substitution methods was distracting (Jimenez and Fishel, 2014; Pylatiuk et al., 2014).

Significant progress has been made toward the development of non-invasive and invasive peripheral and cortical neural interface technologies for providing multiple channels of sensory feedback in a more intuitive manner. Tactors have been used to non-invasively vibrate regions of skin covering tissue that has under gone targeted muscle reinnervation. Impressive subject-specific mappings have been published that show the regions of the chest, for example, that can be stimulated to induce percepts on the phantom limb (Kuiken et al., 2007). Subjective and objective outcome measures have shown that the use of tactors reinforces one’s sense of embodiment of the artificial limb (Marasco et al., 2011).

Peripheral neural interfaces, such as nerve cuff electrodes, have been used to stimulate the median, ulnar, and radial nerves in the residual limb (Navarro et al., 2005). Such electrodes have recently been used to provide simultaneous proprioceptive and tactile feedback to different regions of the phantom limb (Tan et al., 2014a,b). Interestingly, when two distinct channels on the electrode were stimulated simultaneously, subjects reported percepts in regions of the phantom limb that were not previously reported after stimulation by any individual channel. Although further subject-specific characterization of this phenomenon is necessary, it is clear that the provision of simultaneous tactile and proprioceptive feedback will be possible for many more regions of the phantom limb than there are physical neural interface channels. After stimulation sessions, amputees have reported changes in their previously paralyzed phantom limb postures and, importantly, a reduction in phantom limb pain. Some subjects even reported that they were practically pain free (Tan et al., 2014b).

Intracortical microstimulation has been used in brain–machine interfaces for the provision of tactile and proprioceptive feedback (Velliste et al., 2008; Rincon-Gonzalez et al., 2012). In non-human primate studies, electrical stimulation in somatosensory cortex has been used to convey limb movement, although the provision of absolute limb position remains a challenge, irrespective of the neural interface method being used (London et al., 2008). While intracortical microstimulation has been shown to be an adequate tool for influencing the perception of limb motion, stimulation in area 3a also elicits detectable changes in electromyograms in associated musculature (Witham and Baker, 2011). However, the sensations that are elicited by area 3a stimulation remain unknown.

Researchers have also vibrated tendons in the residual limb to provide proprioceptive feedback to amputees. It is hypothesized that the vibrations excite muscle spindles such that a muscle lengthening is perceived. For example, vibration of an extensor tendon can create the sense that the associated joint is being flexed. Amputees have been able to sense joint motion in the phantom limb, as when opening or closing the hand (Marasco, 2014a).

### The “BairClaw” robot hand testbed

**Figure 1:**
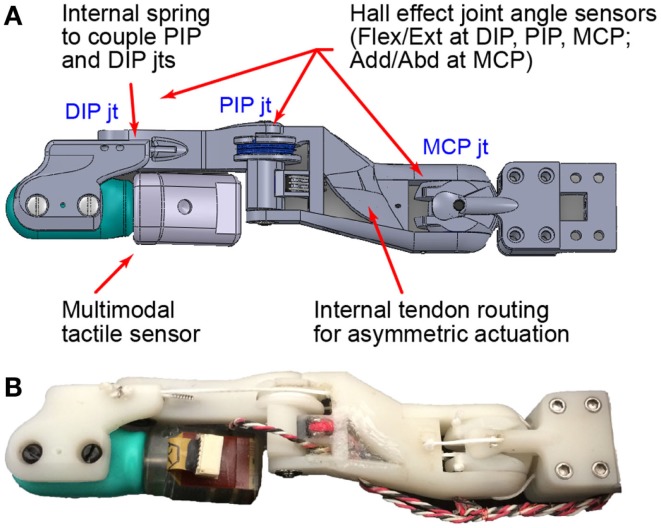
**The BairClaw index finger has four degrees of freedom: DIP, PIP, and MCP flexion/extension and MCP adduction/abduction**. The DIP and PIP joints are passively coupled by a spring. Joint angles are measured by Hall effect sensors while temperature, vibration, and skin deformation are provided by a multimodal tactile sensor. Dorsal views of the **(A)** design schematic and **(B)** prototype are shown.

In this work, we present the “BairClaw,” a highly sensorized, multi-articulating, anthropomorphic robot hand testbed with rich proprioceptive and tactile sensing capabilities (Figure 1). The BairClaw was originally conceived for the advancement of artificial grasp, manipulation, and haptic exploration. We posit that the system could also be used for the neurorehabilitation of somatosensory disorders due to upper limb impairment or loss.

Thus far, efforts to enhance an amputee’s sense of embodiment with prosthesis have focused on cosmetic appearance via the development of five-digit, multi-articulating prosthetic hands and attempts to design realistic skin-like cosmesis (Marasco et al., 2011). We believe that embodiment additionally requires the development of consistent action–perception relationships and their encoding in the nervous system. The BairClaw testbed was designed to enable the development of consistent action–perception relationships that enhance one’s sense of embodiment for robotic or human-in-the-loop use. MVF techniques that facilitate neural plasticity can be further enhanced through the provision of rich proprioceptive and tactile feedback in synchrony with action. By providing amputees with the ability to control, visualize, and feel physical finger–object interactions in a controlled clinical setting, it may be possible to extend current therapies that focus on visualizations of posture alone.

## Materials and Methods

The BairClaw currently consists of an index finger only, but will be further expanded to three and five digits. The modular, tendon-driven, back-drivable design and its artificial proprioception system, and scalable communication system are described in further detail here.

### Remote tendon actuation system

Commercially available multi-articulating artificial hands use an intrinsic actuation system in which motors reside in the palm or at finger joints themselves (Belter et al., 2013; Controzzi et al., 2014). As a result, these hands are either limited in movement speed or grip strength. For the purposes of a testbed, the BairClaw was designed to have an extrinsic, tendon-driven, remote actuation system to enable human-like speeds and grip strengths while maintaining the small volume and form factor of a human-sized hand. The testbed was designed for a maximum fingertip force of 44.5 N (10 lbf) and maximum individual tendon tensions of 111 N (25 lbf). The maximum fingertip force was selected to be consistent with human capabilities for opposition pinch and single-digit force production against a surface (Swanson et al., 1970; Keenan and Massey, 2012). The maximum individual tendon tension was estimated based on planned BairClaw kinematics and overall dimensions. As with the human hand and its extrinsic muscles, the BairClaw’s extrinsic actuation system resides proximal to the wrist and transmits multi-articular joint torques using tendons.

Under load, friction in the tendon routing system can significantly influence the dynamics of the system and cannot be overlooked (Nahvi et al., 1994). Thus, each tendon (200 lbf Spectra line, Power Pro) was routed through a low friction PTFE sheath, four of which were additionally bundled within a polyethylene vacuum line that serves as the supporting structure of the Bowden cable design. Bowden cables consist of an outer sheath that is constrained at both ends while the internal cable transmits a pulling force. The outer cable is flexible and constant in length allowing for force transmission. Additionally, tendon paths were rerouted using small, ball bearing-mounted pulleys.

**Figure 2:**
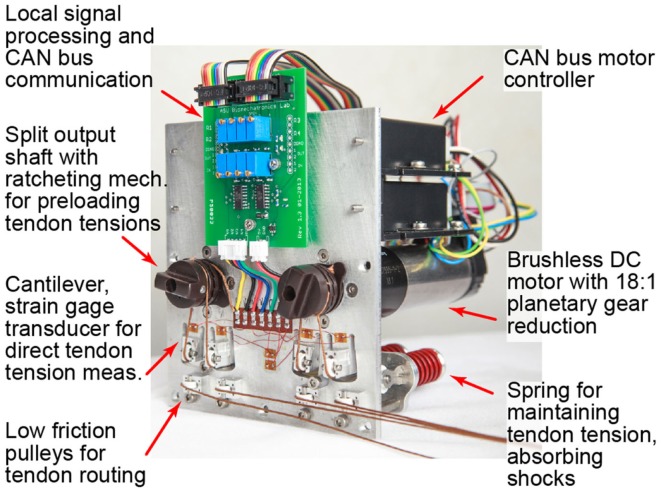
**The modular remote actuation system is shown for two degrees of freedom in an “N-type” configuration: one motor each for PIP/DIP and MCP flexion/extension**. A customizable circuit board locally amplifies and samples tendon tensions for transmission on the CAN bus.

A modular motor bank was used to apply tendon tensions and/or excursions (Figure 2). The design of the motor bank allows for either a “2N-type” or “N-type” set-up. With a 2N-type arrangement, there are two motors per joint allowing for independent control of a flexor and extensor tendon and enabling co-contraction and joint stiffness control. With an N-type arrangement, a single motor is used at each joint in a “push-pull” fashion such that rotation of the motor shaft in one direction flexes the joint and rotation in the other direction extends the joint (Jacobsen et al., 1989). It was desired that the motor bank allow for the actuation of any tendon-driven mechanism. Thus, each motor has a split output shaft with a spring-loaded ratcheting mechanism to allow for quick setup and adjustment of tendon preloads.

Each motor (EC-max 30, 60 W, Maxon Precision Motors, Inc.) is controlled by an EPOS 24/5 controller, which is connected to a CAN bus. Built-in microcontrollers allow for the offloading of low-level processes during position or current control, which aids in reducing the bandwidth of the bus and enables fast communication rates. Since the BairClaw is intended as a testbed, features such as weight, size, and power consumption of the actuation system were not optimized.

### Asymmetric finger design

The BairClaw was designed to accommodate a multimodal tactile sensor (BioTac, SynTouch LLC) that has an immobile distal interphalangeal joint (Fishel et al., 2014). In order to perform complex, human-inspired motions such as stroking and rolling of objects between the fingertips, the distal interphalangeal joint was restored through the use of an asymmetric finger design. The four degree-of-freedom index finger features flexion/extension and adduction/abduction at the metacarpophalangeal joint (MCP) and coupled flexion/extension of the proximal and distal interphalangeal joints (PIP and DIP, respectively). The proximal end of the BioTac can rotate toward and slightly through the dorsum of the hand, thereby allowing the distal joint to function normally (Figure 1B; Video in Supplementary Material).

An embedded spring in the middle phalanx controls the flexure of the DIP joint during PIP and DIP joint flexion. The spring slightly increases the torque required to flex the DIP joint, which causes the PIP joint to flex first. Motion at the DIP joint begins when the PIP joint has reached its full range of motion or if an object impedes PIP joint motion. The spring and an internal PIP joint pulley allow for a low friction, passive compliance of the finger. To minimize friction, all joints were supported by ball bearings, and a PTFE-lined internal channel within the proximal phalanx was used to route tendons.

Eventually, an elastomeric cosmesis could be used to hide the slight protrusion of the BioTac through the dorsal surface of the hand, so as not to break the anthropomorphic illusion for embodiment purposes. However, such illusions are fragile and strengthening of the body schema may be better served by accurate motions and the provision of sensory feedback consistent with actions as opposed to an anthropomorphic appearance only.

### Artificial proprioception system

#### Joint angle measurement

In the index finger prototype, Hall effect sensors were used to measure four sets of joint angles: flexion/extension and adduction/abduction at the MCP joint, flexion/extension at the PIP joint, and flexion/extension at the DIP joint. Each joint angle sensor comprised a Hall effect sensor that measured the change in magnetic field induced by the rotation of a diametrically magnetized ring magnet. Various Hall effect sensor and ring magnet combinations were used to optimize the resolution of each sensor over the full, joint-specific range of motion (Table 1), and were designed to measure joint angles with a resolution of ≤1. It was desired that all proprioceptive sensors be sampled at rates of at least 100 Hz.

**Table 1:** **Specifications for the artificial proprioception, tactile sensor, and remote actuation subsystems**.

	Sampling rates (Hz)	Design range (min, max)	Design resolution
**Bairclaw index finger**
Joint angle sensors
DIP flex/ext	100–1000	(−30°, 90°)	0.12°
PIP flex/ext	100–1000	(−10°, 90°)	0.10°
MCP flex/ext	100–1000	(−30°, 90°)	0.12°
MCP add/abd	100–1000	(−15°, 15°)	0.03°
Multimodal tactile sensor [BioTac, SynTouch LLC Fishel et al. (2014)]
Electrode impedance (19 electrodes total)	100–200 (per elec.)	(0, 3.3 V)	3.2 mV
Internal fluid pressure	100–200	(0, 100 kPa)	36.5 Pa
Vibration	2200–4400	±0.76 kPa	0.37 Pa
Temperature	100–200	(0, 75°C)	0.1°C
Thermal flux	100–200	(0, 1°C/s)	0.001°C/s
**Remote actuation system**
Tendon excursion	200	–	0.9 μm
Tendon tension	200	0, 111 N (25 lbf)	0.11 N (0.025 lbf)

#### Tendon tension measurement

Each tendon was routed over multiple low friction pulleys (Figure 2). A spring-loaded pulley was used to maintain tendon tension and to provide passive compliance for unexpected loads or impacts during operation. For each tendon, another pulley was placed on the end of a cantilever beam cut into the motor plate. In order to measure tendon tensions, the base of each cantilever beam was instrumented with strain gages. A half Wheatstone bridge configuration was used for temperature compensation with reference gages located centrally on the plate. All gage measurements were amplified and sampled locally using a custom circuit board. Trimpots on the board allow for customization of baseline values, amplification, and resolution according to each tendon’s range of operation (Table 1). It was desired that tendon tensions be measured with a resolution of ≤1 N (0.22 lbf).

Tendon tensions are sampled via the EPOS 24/5 motor controllers and transmitted over the central CAN bus. By using the motor controllers to sample tendon tensions, we can scale the entire testbed by simply daisy chaining more controllers onto the CAN bus with little to no modification of the low-level communication scheme.

### Communication and scalability

The BairClaw testbed is controlled by a central, Linux computer running Ubuntu 12.04 that has been modified with a Xenomai kernel patch for hard real-time operation. All communication is performed on a CAN bus, a standard in industrial automation and motor vehicles that ensures real-time communication with simple message packets and a node-based communication structure. Data transmitted via CAN and USB (for the multimodal tactile sensor) are recorded and logged in real-time. Since CAN uses a simple two-line bus, the entire system can easily be scaled by daisy chaining additional digits and motor controllers onto the original bus.

### Controller design

A variety of control schemes could be devised for the testbed. A position controller could use proprioceptive sensor data from joint angle sensors and motor encoders as control signals. A force controller could use proprioceptive sensor data, such as tendon tensions and motor current, or tactile sensor data as control signals. Tendon tensions and moment arms, known from design schematics, can be used to calculate joint torques created by the multi-articular tendons. Standard robotics equations can be applied to relate joint motion to fingertip motion, or joint torque to fingertip forces and torques in three dimensions (Murray et al., 1994).

**Figure 3:**
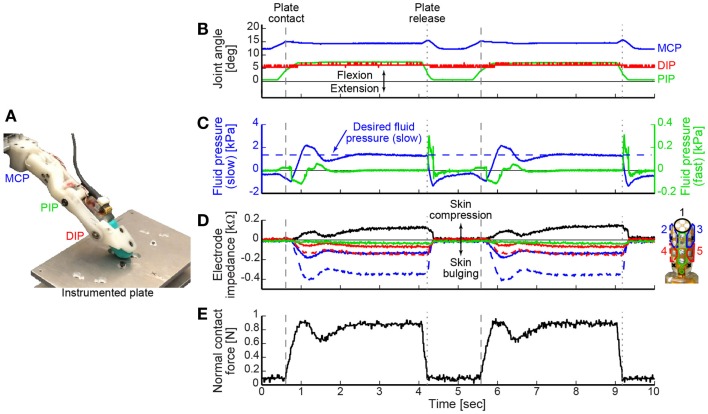
**(A)** The BairClaw was used to perform a tap-and-hold experiment against an instrumented plate. **(B)** Joint angles, **(C)** tactile sensor internal fluid pressure (left *y*-axis) and microvibration (right *y*-axis), **(D)** tactile sensor skin deformation, and **(E)** normal contact force data are shown for two cycles of motion and force production. As the finger flexed and the tactile sensor’s internal fluid pressure exceeded a threshold value, the position controller switched to a force controller designed to achieve and maintain a desired fluid pressure value [horizontal dashed line in **(C)**]. Fingertip contact with and release from the plate are indicated by dashed and dotted vertical lines, respectively.

For demonstration purposes, we illustrate the use of a hybrid position and force feedback controller for a cyclic tap-and-hold task (Figure 3A; Video in Supplementary Material). The controller was designed to function as a state machine that initially operates in position control and moves at a set rate to achieve a commanded posture unless the finger pad comes into contact with an object. In this example, once the tactile sensor’s internal fluid pressure exceeded a threshold, the position controller switched to a force feedback controller designed to achieve and maintain a desired fluid pressure value (as a proxy for fingertip contact force). Specifically, the fluid pressure signal was used in a proportional-integral-derivative feedback controller to quickly achieve and maintain the desired reference value with zero steady-state error.

## Results

### Artificial proprioception system

#### Joint angle calibration

**Figure 4:**
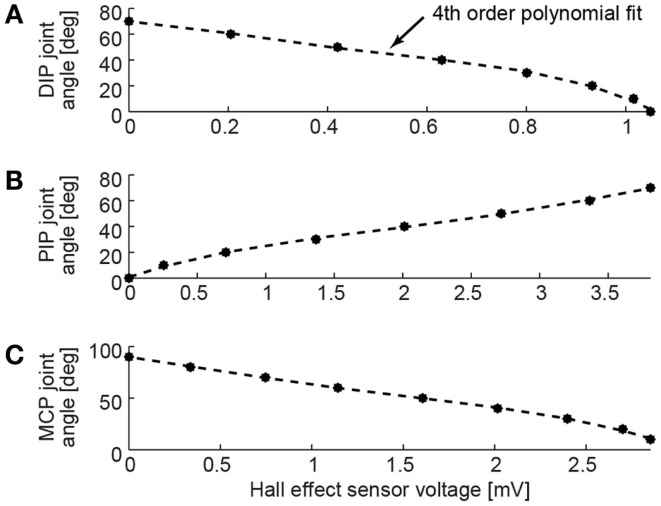
**Joint angle measurement calibrations are shown for the (A) DIP, (B) PIP, and (C) MCP flexion/extension degrees of freedom**. Fourth order polynomial fits performed on calibration data collected in 10° increments resulted in *R*^2^ > 0.99 for each joint. Positive angles indicate joint flexion from a neutral position at 0°.

The joint angle measurement system was calibrated for each joint’s range of motion in 10° increments using a goniometer. The Hall effect sensors were designed to respond linearly to changes in the magnetic field. Due to small variations of the magnetic field near the ends of the range of motion, it was necessary to fit a fourth order polynomial model to the sensor response (Figure 4). Each model had a coefficient of determination *R^2^* value >0.99. The slope of the calibration curves depended on space requirements within the limited volume of the BairClaw finger and the Hall effect sensor configuration at each joint.

#### Tendon tension calibration

The tendon tension measurement system was calibrated using a multi-step process that accounted for interactions between neighboring strain gages on the motor plate. In general, the change in resistance of a strain gage is linearly dependent on the internal strain and stress at the location of the sensor. Due to highly sensitive gages and the close proximity of pulleys on the motor plate, each half Wheatstone bridge sensed strain caused by tendons routed over nearby pulleys.

**Figure 5:**
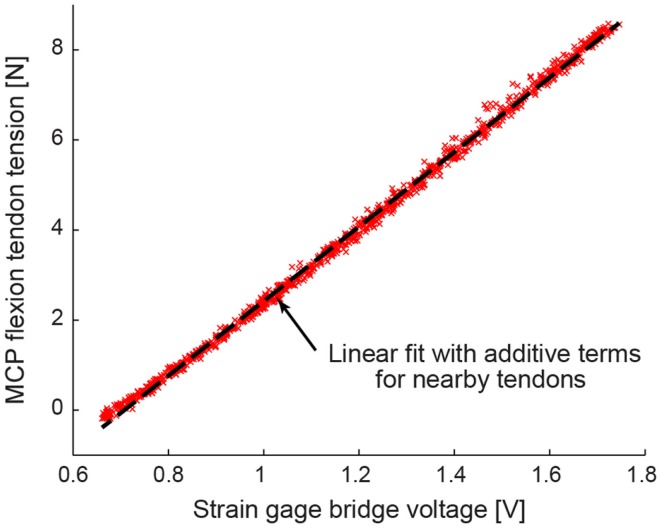
**A representative tension calibration is shown for the MCP flexion tendon**. Using 1000 trials of randomly selected tendon tension combinations, each tendon tension sensor was fit to a linear model that comprised a sum of scaled tensions of tendons mounted nearby on the motor plate.

A custom calibration rig was built that randomly applied a known force to all four tendons simultaneously. One thousand trials of randomly selected tendon tension combinations were applied, with each individual tendon tension ranging from 0 to 50 N. Each tendon tension sensor was fit to a linear model that comprised a sum of scaled tensions of tendons mounted nearby on the motor plate (Figure 5). The calibration models were cross-validated using a Lasso method to minimize mean squared error. The Lasso method returned comparable models and confirmed the statistical significance of the additive terms associated with nearby tendons (Tibshirani, 1996).

### Hybrid position and force feedback controller

The tap-and-hold example demonstrates the speed with which the BairClaw testbed can switch control modes, the versatility of control using a variety of feedback control signals, and the stability of the overall mechatronic system (Video in Supplementary Material). Figures 3B–E shows two cycles of a tap-and-hold experiment in which joint angles and angular velocities were tracked as the BairClaw flexed to a pre-specified posture. Before the final posture could be achieved, the BairClaw fingertip contacted a plate instrumented with a six degree-of-freedom load cell (Nano-17, ATI Industrial Automation). Once the internal fluid pressure of the tactile sensor reached a threshold, the position controller switched to a force controller to achieve and maintain a desired reference fluid pressure value. The pre-specified posture and fluid pressure threshold and reference values were selected arbitrarily for demonstration purposes, but could be set according to the context of the experimental task.

Figures 3B–D show the joint angles, tactile sensor internal fluid pressure, microvibration, and skin deformation data. As with any higher order, underdamped system, a slight overshoot occurred in the internal fluid pressure control signal, but was quickly corrected (Figure 3C). Trends in the normal contact force measured by the instrumented plate aligned with the internal fluid pressure signal used for force feedback control (Figure 3E).

The tactile sensor’s electrode impedance values provide information on skin deformation caused by the BairClaw’s forceful interaction with the plate. Individual electrode impedance values were grouped into anatomically meaningful clusters for visualization purposes. Increases in impedance indicate that the skin is being compressed toward the sensor’s rigid core while decreases in impedance indicate bulging away from the core. As expected, the electrode impedance data indicate compression of the skin at the fingertip and bulging of the skin on other regions of the finger, especially in the distal regions, during the tap-and-hold phase of the trial.

In order to relate one’s voluntary actions to resulting stimuli (visual or otherwise), there must be minimal delay between the action and the perceived stimuli. Previous work has shown that delays for myoelectric prosthetics should be kept below 125 ms (Farrell and Weir, 2007). The majority of the delay found in myoelectric controllers is due to processing of the myoelectric signals. The BairClaw is capable of processing and reacting to various inputs within a single sampling period (10 ms). Mechanical and computational delays, estimated from the delay between the switching of the controller and a measurable change in system response, were approximately 65 ms in the tap-and-hold example. Any additional delays for a human-in-the-loop configuration would be specific to the human–machine interface and signal processing, such as pattern recognition that may be performed on the human command signals.

## Discussion

### Availability of sensor technology in artificial hands

To date, the availability of proprioceptive sensors in commercially available prosthetic hands has been limited (Controzzi et al., 2014). Joint angle encoders are not available in commercially available myoelectric prostheses, rather motor encoders are used to estimate grip aperture. However, motor encoders cannot be used to estimate the posture of multi-articulating digits when underactuated finger designs are used. For instance, the tendon-driven, conformal grasp of prosthetic hands such as the Touch Bionics i-limb prosthesis (Touch Bionics I-Limb Product Range, 2014) reduces the degrees of freedom that an amputee must consider for control, but specific hand configurations cannot be measured or conveyed to an amputee in real-time. One recent study with the i-limb reports the use of motor current monitoring and timing of finger movements from the i-limb’s neutral, fully opened position as a way to estimate joint angles (Kyranou, 2014). Future work is required to overcome limitations resulting from assumptions about finger velocity, battery power, and object rigidity. The VINCENT hand prosthesis (Vincent Systems VINCENTevolution 2, 2014) was designed with less joints per digit than the biological hand and a spring is used to couple joints in each digit. In a research model of the VINCENT hand called the “Bionic Hand,” flex/bend sensors were placed at the metacarpal joints only (Vincent Systems Bionic Hand, 2014). The rigid link design of the RSL Steeper Bebionic hand prosthesis (Medynski and Rattray, 2011; RSL Steeper Bebionic3 Hand, 2014) facilitates the use of motor encoders to track digit posture, but the system does not currently include tactile sensors.

Tactile sensor technology is also scarce in commercially available prosthetic hands, and remains unimodal in nature (Controzzi et al., 2014). The one degree-of-freedom Otto Bock SensorHand Speed hand prosthesis (Puchhammer, 2000) uses fingertip sensors to detect the slip of a grasped object. Some commercially available myoelectric hands have been modified with multimodal tactile sensors as research tools (Jimenez and Fishel, 2014). Advanced multi-articulating prosthetic hands produced by the DARPA Revolutionizing Prosthetics Initiatives 2007 and 2009, such as the DEKA “Luke Arm” and the Johns Hopkins University Applied Physics Lab “Modular Prosthetic Limb” (Otto, 2013), are highly sensorized, but access to these systems remains limited.

Since affordable, off-the-shelf solutions were unavailable, the BairClaw testbed was designed to be highly sensorized for both proprioception and multimodal tactile sensation from the ground up. Joint angles are measured directly at each joint and with minimal drift. As a result, the BairClaw proprioception system enables accurate joint angle tracking without having to cycle the hand through neutral postures to reset postural baselines, as with commercially available prosthetic hands. The proprioception system utilizes inexpensive ring magnets and Hall effect sensors to achieve joint angle resolution of just over a 10th of a degree at 100–200 Hz sampling rates. The BioTac is capable of measuring multiple types of graded tactile information at data rates of 100–4400 Hz. The BairClaw testbed is limited in that the system requires tethered power and users cannot don the bulky actuation system. While the entire testbed was not designed to be donned by subjects, the hand itself can be mounted to a lightweight test socket for whole arm experiments. The inclusion of rich proprioceptive and tactile sensing will enable the study of action–perception relationships, the development of new feedback control schemes, and the ability to provide amputees with simultaneous proprioceptive and tactile sensory feedback via cutting edge neural interface techniques.

### Applications of the BairClaw testbed to neurorehabilitation of the body schema

The hope that a mechanical or robotic system could become part of one’s body schema is hardly a new idea. MVF and GMI techniques are established methods for the manipulation of body schema through visual feedback alone. The use of MVF is an important paradigm shift in the treatment of neurological damage to the brain and peripheral nervous system, as the technique seeks to take advantage of the dynamic restructuring capabilities of the brain to manipulate body schema. It is believed that the illusory influence of visual feedback can be further enhanced by simultaneous proprioceptive and tactile feedback that is congruent with what is being seen.

#### Closing the somatosensory loop with amputees

While the development of neural interfaces for proprioceptive and tactile feedback is hardly a solved problem, promising new techniques and highly encouraging findings are being reported. For instance, intrafascicular multichannel electrodes inserted into median and ulnar nerves have been used to provide real-time sensory feedback of a bidirectional prosthetic hand (Raspopovic et al., 2014). Force sensors at prosthetic fingertips were used to drive electrode stimulation currents. The amputee subject was able to exploit features of the dynamic, graded tactile feedback, such as rates of change of current amplitude and differential timing of contact across the hand, to distinguish between objects based on stiffness and shape, respectively.

More recently, researchers have used selective, non-penetrating peripheral nerve cuff electrodes to stimulate residual upper limb nerves in unilateral amputees (Tan et al., 2014b). Using a systems identification approach, they were able to elicit long-term stable, graded, natural percepts including tapping, constant pressure, vibration, and even light moving touch, all of which could be driven by a highly sensorized testbed, such as the BairClaw. Percept area and intensity could be modulated via stimulation intensity and frequency, respectively. Percept sites were numerous, independent, well-defined, and even included sites on fingertips. Proprioceptive percepts remain to be systematically explored and mapped.

Using closed-loop feedback, subjects were able to accomplish dexterous tasks while blindfolded. In addition to the functional benefits enabled by the sensory system, there were positive embodiment-related and therapeutic effects as well. According to page 9 of Tan et al. (2014b), “When sensation was active, both subjects perceived the hand and prosthetic hand to be nearly perfectly colocated in space. When sensation was not active, the prosthesis was viewed by the subjects as a tool that extended beyond their hands.” Although further investigation is required, it is exciting that both subjects reported the elimination of phantom limb pain with the use of the sensory feedback system.

Other researchers have recently used a non-invasive tendon vibration technique to elicit percepts of joint-specific movement via the “Kinesthetic Illusion” effect (Marasco, 2014a,b). It is well-known that tendon vibration creates an illusion of muscle lengthening (Lackner, 1988). Working with amputees who had undergone targeted sensory reinnervation, researchers were able to vibrate reinnervated muscle to produce the percepts of different, gross hand postures, including a cylinder grip, precision pinch, and opening of the hand.

While methods for communication between artificial hands and the human nervous system continue to improve, further investigation is needed to address gaps that remain. For instance, a myriad of high resolution joint angles can easily be obtained from highly sensorized artificial testbeds, such as the BairClaw. However, it is unclear how to convey this detailed postural information via coarse methods for artificial proprioceptive feedback such as tendon vibration, which has been recently used to convey a small number of gross hand postures. Furthermore, the lack of validated, objective functional outcome measures for upper limb myoelectric prosthesis use, let alone bidirectional prosthesis use, makes it difficult for researchers to relate the quality of artificial sensory feedback to improvements in quality of life (Wright, 2006; Hill et al., 2009).

#### Extending vision-based therapies with a high-tech rubber hand illusion

The high occurrence of phantom limb pain and proprioceptive disorders may be due to the lack of embodiment, or a disrupted sense of ownership due to mismatches between different modalities of sensory feedback, such as touch and vision (Harris, 1999). Prior efforts to improve the embodiment of prosthetic devices have focused on appearance. Although visual appearance is extremely important to amputees for both embodiment and interactions with others, it is a fragile illusion. Surveys have shown that sensory feedback is often ranked as a higher priority than life-like appearance for powered prostheses (Biddiss and Chau, 2007b; Biddiss et al., 2007). Through artificial proprioceptive and exteroceptive feedback to a phantom limb, an amputee could develop and maintain an internal model of a neuroprostheses as part of his/her body schema.

It has already been reported that phantom limb pain could be reduced when sensory substitution via electrotactile stimulation was used to provide feedback on grip force to transradial amputees. Clinically relevant improvements were observed even after a short 2 week training period (Dietrich et al., 2012). It has been postulated that, based on the increased functionality and decreased phantom limb pain that was observed, a cortical reorganization likely occurred (Elbert, 2012). Thus, it may be possible to rehabilitate body schema and reduce chronic pain through the dynamic restructuring of neuronal pathways in the brain, even with sensory substitution techniques.

In this work, we demonstrated the use of the BairClaw testbed for a tap-and-hold experiment that resembles a task that might be done in an MVF therapy session. The BairClaw testbed has also been used to produce stroking motions for haptic exploration of surfaces (Video in Supplementary Material) and to track fingertip forces from a non-human primate precision grip task (Hellman et al., 2014). Preliminary results suggest that the BairClaw is capable of fine fingertip force control at physiologically meaningful magnitudes and timescales.

## Conclusion

Established therapeutic techniques such as MVF and GMI rely purely on visual feedback or imagined action, respectively. The rubber hand illusion demonstrates the power of visual feedback combined with somatosensory feedback. Advances in artificial hand technology and techniques for providing proprioceptive and exteroceptive feedback now make it possible to combine MVF with a high-tech version of the rubber hand illusion. The efficacy of visual manipulations for neurorehabilitation could be enhanced if coupled with proprioceptive and tactile feedback in a controlled therapy environment.

Even at this nascent stage of neural interface development, reports of natural proprioceptive and tactile percepts from upper limb amputees are highly encouraging. It will soon be possible to provide graded, natural percepts to amputees that could be driven directly by joint angle encoders, tactile sensors, skin stretch sensors, thermistors, etc. There are numerous potential benefits of enhanced embodiment by way of congruent multisensory feedback that is spatiotemporally consistent with commanded actions: reduction of phantom limb pain, a renewed sense of ownership, stronger connections to others and to society, and increased use of prosthesis due to improved functionality and reduced cognitive burden. We believe that highly sensorized testbeds such as the BairClaw can be used to enhance the embodiment of a neuroprosthesis into one’s body schema, and be used to probe the complex relationships between sensory feedback, illusion-based percepts, and body schema manipulation.

## Conflict of Interest Statement

The authors declare that the research was conducted in the absence of any commercial or financial relationships that could be construed as a potential conflict of interest.

## Supplementary Material

The Supplementary Material for this article can be found online at http://www.frontiersin.org/Journal/10.3389/fnhum.2015.00026/abstract
